# Validation and Psychometric Properties of the Tobacco Urge Management Scale (TUMS)

**DOI:** 10.3390/ijerph20085453

**Published:** 2023-04-10

**Authors:** Samantha M. Chin, Stephen J. Lepore, Bradley N. Collins, Levent Dumenci, Maria A. Rincon

**Affiliations:** 1 Department of Social and Behavioral Sciences, Temple University, Philadelphia, PA 19122, USA; samantha.davis@temple.edu (S.M.C.); collinsb@temple.edu (B.N.C.); maria.rincon@temple.edu (M.A.R.); 2 Department of Epidemiology and Biostatistics, Temple University, 1301 Cecil B. Moore Avenue, 9th Floor, Ritter Annex, Philadelphia, PA 19122, USA

**Keywords:** smoking, underserved populations, smoking abstinence, smoking urge management

## Abstract

Background: During quit attempts, smokers must overcome smoking urges triggered by environmental cues and nicotine withdrawal symptoms. This study investigates the psychometric properties of the 12-item Tobacco Urge Management Scale (TUMS), a new measure of smoking urge management behaviors. Methods: We analyzed secondary data (*n* = 327) from a behavioral smoking cessation intervention trial, Kids Safe and Smokefree (KiSS). Results: Confirmatory factor analysis of the TUMS indicated that a one-factor model and a correlated two-factor model had similar model fit indices, and a Chi-square difference test supported the one-factor model. Further study of the parsimonious one-factor scale provided evidence of reliability and construct validity. Known group validity was evidenced by significantly higher TUMS scores in the KiSS intervention arm receiving urge management skills training than in the control arm (*p* < 0.001). Concurrent validity was evidenced by TUMS’s inverse association with cigarettes smoked per day and positive associations with nonsmoking days, 7-day abstinence, and self-efficacy to control smoking behaviors (*p*’s < 0.05). Conclusion: The TUMS is a reliable, valid measure of smoking urge management behaviors. The measure can support theory-driven research on smoking-specific coping mechanisms, inform clinical practice by identifying coping strategies that might be under-utilized in treatment-seeking smokers, and function as a measure of treatment adherence in cessation trials that target urge management behaviors.

## 1. Introduction

Tobacco smoking remains among the top global public health threats, claiming more than eight million lives yearly [1]. Cigarette smoking is the most common form of tobacco consumption, although some populations prefer products such as tobacco waterpipes, cigars, and bidis [2]. Quitting smoking is challenging for most smokers, primarily due to difficulty overcoming smoking urges associated with nicotine withdrawal. Cognitive-behavioral therapy (CBT)-based interventions routinely incorporate urge management skills training to help smokers quit [3]. Successful interventions address cognitive and behavioral skills to help smokers manage urges in different situations [4]. Currently, few intervention studies assess smokers’ use of urge management strategies. Thus, it is unclear whether interventions are effective at promoting urge management, the extent to which participants adhere to recommendations, or how urge management skills might improve smoking outcomes. A valid and reliable measure of smoking urge management would help address these gaps. To this end, investigators of the Kids Safe and Smokefree (KiSS) trial [5,6,7] created the Tobacco Urge Management Scale (TUMS), a self-report measure of smoking urge coping behaviors. The measure was initially designed to assess intervention treatment adherence (i.e., whether intervention participants used recommended smoking urge management strategies) and identify mechanisms of action (i.e., whether urge management strategies helped smokers moderate their smoking). However, the TUMS also has value in theory testing and clinical practice. This paper reports on the TUMS’s dimensionality, validity, and reliability.

The experience of urges to smoke during periods of abstinence is a hallmark of nicotine dependence [8]. Urges are typically characterized as a desire to smoke, often in anticipation of relief from nicotine withdrawal symptoms [9]. Associative learning principles help explain how the repeated pairing of stimuli during nicotine dosing (e.g., smoking) leads to conditioned learning [10]. A wide range of conditioned stimuli, or triggers, are embedded throughout a smoker’s routine and across interactions within their social networks and in settings where smoking is permitted [11]. This conditioned learning contributes to ongoing nicotine dosing during dependence periods [12] and endures after a quit attempt.

CBT-based interventions include skills training to help smokers anticipate urge-eliciting triggers and exert compensatory urge management strategies during a quit attempt. Behavioral and social-cognitive theory conceptualizations of cessation and relapse have guided treatment models that emphasize a transitional behavior change process during a quit attempt. For example, Marlatt’s model of relapse is guided by evidence that effective CBT-framed coping responses used for relapse prevention can enhance self-efficacy to maintain abstinence, which, in turn, reduces relapse [13,14]. This process recurs through repeated exposures to urge-eliciting conditioned stimuli across multiple contexts for months after initiating a quit attempt [15].

Extensive evidence [16,17,18,19,20] has established the effectiveness of CBT-based coping skills training to overcome urges to smoke during quit attempts. For example, Shiffman and colleagues [21,22] found that former smokers’ use of cognitive and behavioral coping strategies to manage urges was integral to maintaining abstinence. Perri and colleagues [23] showed the benefits of behavioral self-control coping strategies among self-quitters. Prochaska, DiClemente, and colleagues [24,25] have also suggested the importance of coping strategies during various phases of a quit attempt. 

Our conceptualization of coping skills for urge management in the KiSS intervention [5,6,7] was guided by Shiffman’s work [21], effective CBT techniques for coping skills training in smoking interventions, and Marlatt’s [13] conceptual model of relapse. For example, our skills training approach included telehealth counselors educating participants about cognitive and behavioral coping skills for relapse prevention (e.g., avoiding or escaping situations that elicit urges to smoke and distracting oneself from urges with compensatory thoughts and activity). The TUMS measure reflected the intervention strategies. 

Much smoking cessation research does not investigate urge-specific coping. Instead, it examines how smokers’ general approach to coping with stressors affects cessation and relapse. The evidence linking general coping behaviors to successful smoking control is weaker than that linking smoking-specific coping and smoking control. For example, one study [26] found that smokers’ strategies for coping with life stressors, as measured by the Brief COPE scale [27], did not predict smoking cessation. In contrast, Carey and colleagues [28] asked smokers contemplating quitting to think about their efforts to quit smoking while completing a general life-stress coping measure. They found that relative to nonquitters, quitters reported greater use of problem-solving and cognitive restructuring and less wishful thinking, self-criticism, and social withdrawal. These latter findings suggest that a measure designed specifically for coping with the challenges of quitting might be more predictive of smoking outcomes than a non-specific coping measure.

We found one measure that explicitly focused on coping with urges to smoke, the 3-item Coping with Temptation to Smoke (CTS) scale [29]. The CTS is part of a larger multidimensional measure of smokers’ self-change strategies. It has modest reliability (Cronbach’s alpha = 0.73) and limited validity evidence. For example, Etter and colleagues [29] noted that the CTS items have face validity, but the scale only captures one type of urge coping—distraction—and does not predict smoking cessation. Another study by Etter and colleagues [30] showed higher CTS scores (i.e., greater distraction urge coping) among males who quit versus those who did not but found no such differences among females. A third study showed no association between CTS scores and smoking abstinence [31]. While the CTS is specific to coping with smoking urges, its modest reliability and exclusion of a range of cognitive and behavioral coping strategies for coping with urges are limitations. 

The TUMS measure addresses the need for a theoretically grounded, valid, and reliable measure of how smokers specifically cope with the urge to smoke. The measure could be used to evaluate treatment adherence in interventions targeting urge management behaviors and advance theoretical models of successful smoking cessation treatments. For example, O’Connell and colleagues [32] have noted that a valid measure of urge management could be used to discover whether urge management behaviors facilitate cessation and relapse prevention by lowering urge levels or making the urges more tolerable. Concerning treatment adherence, smokers’ scores on the measure could alert interventionists about the acceptability and uptake of recommended coping strategies and inform future intervention improvements. A measure of client adherence to urge management behavioral recommendations would also be valuable in a cessation counseling practice. For example, a cessation counselor could use the TUMS to measure treatment progress or evaluate clients’ repertoire of urge management strategies and encourage them to experiment with the wide range of strategies captured by the measure.

## 2. Methods

### 2.1. Overview of the KiSS Trial 

The data for this study were collected in the context of a two-group randomized controlled trial, referred to as KiSS. The Temple University Institutional Review Board approved the trial before any data collection. The KiSS trial evaluated the efficacy of a novel multilevel intervention to reduce child tobacco smoke exposure and parental smoking in low-income and predominantly minority communities [5,6,7]. Tobacco smoke exposure is a preventable cause of morbidity and mortality in children [33,34,35,36,37], and the primary source of exposure is a parent who smokes [38]. We targeted parental smokers in low-income and minority communities because children in such communities are at exceptionally high risk for tobacco smoke exposure [39]. 

The KiSS participants were smoking parents/guardians recruited from safety-net pediatric clinics. All the participants received a minimal intervention, “Ask, Advise, and Refer” (AAR), during a pediatric clinic visit with their child. Pediatric providers asked the parents about their child’s exposure to tobacco smoke at home, advised them about the harm to children’s health and the benefits of protecting children from tobacco smoke, and referred smoking parents to smoking cessation services. After the AAR intervention, eligible and consented smokers were randomized into two potential treatments: additional telephone-based behavioral counseling around child secondhand smoke exposure reduction and parental cessation (AAR + counseling) or nutrition education (AAR + control). The AAR + counseling group received 3 months of telephone-based behavioral counseling that included, among other things, skills training to manage urges to smoke and reduce their child’s secondhand tobacco smoke exposure. The skills included compensatory cognitive and behavioral coping strategies to avoid smoking and child exposure. These skills were introduced as either “things you think of or tell yourself that can help you avoid smoking when you have an urge” (e.g., cognitive skills) or “things you can do when you have an urge to help avoid smoking” (e.g., behavioral skills). The counselors provided the participants with support and problem-solving around smokers’ efforts to use urge management skills throughout the intervention. The AAR + control group received 3 months of telephone-based nutrition education (without any smoking cessation-related skills training).

### 2.2. Measures

Structured, computer-assisted telephone interviews were used to collect self-report data at baseline (before randomization and intervention) and at 3-month end-of-treatment (EOT). 

*Descriptive measures.* The demographic and pre-intervention smoking characteristics were assessed at baseline.

*Tobacco Urge Management Scale (TUMS).* The 12-item TUMS was administered at the EOT. The participants reported how often (1 = never, 2 = rarely, 3 = sometimes, 4 = often) in the past week they used each coping strategy to prevent smoking a cigarette during an urge (see Table 1). The item scores were summed for a frequency of use score. The scale items were based, in part, on Shiffman’s [21,22] model for relapse prevention coping skills. This model has endured based on evidence that combining cognitive and behavioral coping responses effectively delays and prevents relapse. In a follow-up study to Shiffman’s seminal work, O’Connell and colleagues [40] used real-time Ecological Momentary Assessment methods to examine how abstaining smokers cope with urges. Over 10 days, O’Connell et al. found that 67% of the coping responses were behavioral and 33% were cognitive. She also identified subtypes of responses within these broader categories (see p. 492 in [40]). O’Connell’s categories guided us in designing the cognitive and behavioral TUMS items. 

As shown in Table 1, the cognitive urge management strategies included mental distraction (item 1), self-encouragement (items 2 and 4), and thinking of the adverse effects of smoking (item 3). The behavioral strategies included stimulus control (items 5 and 6), substitution with food (item 8), behavioral distraction (item 11), and breathing exercises (item 12). We added three additional behavioral strategies based on relapse prevention intervention research, including delaying smoking to practice urge management (item 7), seeking support (item 9), and self-reward for successful practice (item 10). 

*Indicators of smoking behavior at the three-month end-of-treatment (EOT) period.* The following smoking behaviors were assessed at the EOT: seven-day point prevalence abstinence from smoking, average cigarettes smoked per day in the prior week, the number of nonsmoking days from baseline to EOT, smoking cessation self-efficacy, and smoke-free home/car self-efficacy. The timeline follow-back method via structured interview [41] was used to measure the average number of cigarettes smoked per day in the previous week and the number of nonsmoking days between the baseline and EOT. The 12-item self-efficacy for avoiding smoking scale (α = 0.89) was used to assess smoking cessation self-efficacy at the EOT [42]. The participants rated how sure (1 = not at all to 4 = very) they were that they could avoid smoking in various situations (e.g., over coffee). The response scores were summed, with the higher scores indicating higher self-efficacy to abstain from smoking. Three similar items were created focusing on the efficacy to maintain a smoke-free home/car [7]. The participants reported how sure (1 = not at all to 4 = very) they were that they could “create a smoke-free home/car,” “keep home/car smoke-free,” and “keep child away from other smokers’ tobacco smoke.” The response scores were summed, with the higher scores indicating higher self-efficacy in maintaining a smoke-free home/car.

### 2.3. Analytic Approach

Confirmatory factor analysis (CFA) was used to test the factor structure of the TUMS items under two possible models: A one-factor model of coping skills (factor 1) and a correlated two-factor model of cognitive coping skills (factor 1) and behavioral coping skills (factor 2). Mplus 8.1(Muthén & Muthén: Los Angeles, CA, USA) [43] was used for the analyses, with no additional adjustment to the structural models before analysis. CFA analysis used the mean-variance-adjusted weighted least squares (WLSMV), a robust method to fit polychoric correlations appropriate for ordered, categorical items [44]. The model fit was evaluated using three indices: the comparative fit index (CFI ≥ 0.95), the Tucker-Lewis index (TLI ≥ 0.95), and the Root Mean Square Error of Approximation (RMSEA ≤ 0.07) [45]. In addition, we considered model parsimony as a criterion for the best model fit, meaning that when two models have the same fit values, it is best to select the simpler model (i.e., the model with the smaller number of free parameters). The Chi-square difference test was used to test the null hypothesis that the two models fit equally well. We used standardized factor loadings in assessing the relationship between the latent factors and individual items in the TUMS. 

After identifying the best TUMS model based on CFA, the reliability of the TUMS was estimated using McDonald’s coefficient Omega [46]. McDonald’s Omega was selected over Cronbach’s alpha because it uses model parameters and overcomes some limitations of Cronbach’s alpha [47]. A high Omega (>0.70) and acceptable inter-item correlations (> 0.30) are needed to establish reliability based on internal consistency [48]. 

The TUMS validity was further examined by assessing the relationship of the TUMS scores with the intervention conditions and smoking-related outcomes using IBM SPSS software (Version 26). An independent sample *t*-test was used to test the intervention and control groups’ mean differences in urge management coping. The known group validity of the scale would be evidenced if the TUMS scores were significantly higher in the intervention group than in the control group. Spearman’s correlations were used to examine the associations between the TUMS and average daily cigarettes smoked, the number of nonsmoking days, smoke-free home/car self-efficacy, and smoking cessation self-efficacy. The concurrent validity of the scale would be evidenced by the TUMS scores being significantly and negatively correlated with the average daily cigarettes smoked, and significantly and positively associated with the number of nonsmoking days, smoke-free home/car self-efficacy, and smoking cessation self-efficacy. Concurrent validity was also assessed by testing the mean differences in urge management coping between the participants reporting any versus zero cigarettes smoked (i.e., abstinence) in the prior week using an independent sample *t*-test. Concurrent validity would be evident if the abstainers had significantly higher TUMS scores than the non-abstainers.

## 3. Results

### 3.1. Demographic Characteristics

The participant characteristics are shown in Table 2. The participants (*n* = 327) were primarily African American, female, and between the ages of 18 and 65, and most were under 35 years. The majority had below college-level education and reported living under the poverty level. For the comparison of the model fitness, 29 cases were excluded due to missing information on each of the TUMS items due to attrition at the EOT. Comparisons of the baseline demographic characteristics of the participants with and without missing data revealed no statistically significant differences.

### 3.2. Model Selection

The one-factor model fit indices were RMSEA = 0.075, CFI = 0.962, and TLI = 0.953. The correlated two-factor model fit indices were RMSEA = 0.074, CFI = 0.963, and TLI = 0.953. The analysis showed a difference in RMSEA values of 0.001, indicating both models have a good fit with similar scores. The CFI values indicated a good fit in the one-factor and correlated two-factor models, with a 0.001 difference between the scores. The TLI values between the models were identical, indicating a good fit for both models. The factor correlation included unity (0.905–1.001), indicating a lack of discrimination between the two traits. Furthermore, the Chi-square difference test was not significant, with a Type I error rate of 0.01, confirming that the models fit equally well. Following the parsimony criteria, preference for model selection is given to the simpler one-factor model, with the least free parameters that still meet the goodness-of-fit criteria [49]. 

### 3.3. Standardized Factor Loadings

Table 1 contains the standardized factor loadings for each TUMS item. All the factor loadings were statistically significant (*p* < 0.001), ranging from 0.578 to 0.761. The differences in the factor loading scores suggest that certain items were more strongly associated with the general factor than others. Based on these values, item 2 (“I reminded myself of my goals for a healthier lifestyle for my family and myself”) had the strongest association and item 1 (“I mentally distracted myself from my craving, for example, I meditated, prayed, or listened to music”) had the weakest association to urge coping. 

### 3.4. Reliability and Validity of Single-Factor TUMS

The 12-item measure is reliable, with high internal consistency (McDonald’s Omega = 0.87). To further examine validity, we compared the TUMS scores of the participants in the different intervention conditions. Those in the AAR + counseling intervention condition scored significantly higher on the TUMS than those in the AAR + control condition (M = 33.30, SD = 6.44 vs. M = 29.07, SD = 9.75; *p* < 0.001). Across both groups, the participants who used urge management skills more frequently reported significantly more nonsmoking days, lower cigarettes smoked per day, higher self-efficacy to create and maintain a smoke-free home/car, and higher self-efficacy to abstain from smoking (see Table 3). Consistent with the correlational data, the participants who reported abstaining from smoking for the previous seven days had significantly higher TUMS scores than their counterparts who continued to smoke (M = 2.809, SD = 0.77 vs. M = 2.54, SD = 0.69; *p* < 0.05).

## 4. Discussion

The present study supports the reliability and validity of the TUMS in a population of predominantly low-income and racial minority smokers. The model fit indices for the one-factor and correlated two-factor models indicated both were a good fit. Given the minor differences in the fit indices between the one-factor and correlated two-factor models and the high inter-factor correlation, the one-factor model is preferred for parsimony. The Omega was >0.70, indicating good scale reliability. The higher TUMS scores in the intervention group that received urge management skills training compared with the controls is evidence of known group validity and shows that the TUMS can be used to measure treatment adherence and intervention effectiveness. Concurrent validity was evidenced in multiple ways. Relative to the participants with lower TUMS scores, those with higher TUMS scores had higher levels of self-efficacy to abstain from smoking and maintain a smoke-free home/car, reported more smoke-free days, and smoked fewer cigarettes. The TUMS scores were also higher among the smokers who had quit for at least seven days versus those who had not. These findings suggest that the TUMS is a valid and more useful measure of coping with smoking urges than brief scales, such as the CTS [30], and general measures drawn from the stress and coping literature e.g., [27]. 

A review of the standardized factor loadings across the items suggests that none had weak associations with the latent factor [50]. Nevertheless, the differences across these values indicate that each item is unlikely to contribute equally to the general coping factor. Item 2 had the highest correlation to the latent factor. This item reflects a cognitive strategy of remembering goals for a healthier lifestyle for family and self when a smoking urge arises. The KiSS intervention leveraged parents’ motivation to protect their children’s health by eliminating tobacco smoke exposure and to be available to their children by remaining smoke-free and healthy [5,6,7]. This motivation appears to be an essential factor in resisting smoking urges in this study. In smoking cessation interventions not levered to family health, it is possible that other strategies would be more central. Indeed, item 11, which had a factor loading nearly as high as item 2, is a behavioral distraction strategy (e.g., keep hands busy, go for a walk) that could be effective even among people without children. These findings illustrate the value of using a measure such as the TUMS, with its various urge-coping strategies, rather than a narrow measure, such as the 3-item CTS scale [29].

Determining the best-fit model for the TUMS has conceptual implications. As a single-factor construct, our results suggest that the concept of smoking urge management, as assessed by the TUMS, can be described as a single mechanism for managing urges using a combination of cognitive and behavioral strategies. These findings suggest that interventions should introduce smokers to various urge management strategies, allowing them to tailor their response to the situation and personal preferences. Tools that help smokers easily remember strategies may further support their coping efforts. For example, in the KiSS intervention, we introduced smokers to the acronym “DEADS”—delay, escape, avoid, distract, and substitute—to help them remember key urge management strategies that can help them “slay” their tobacco dependence. The items on the TUMS map onto these key behaviors.

### Strengths and Limitations

The study had several strengths. First, it used a theoretically grounded and evidence-based conceptual framework for developing the TUMS. Second, we intentionally kept the scale brief, so it is a low-burden, practical assessment tool for clinical interventions and research on smoking cessation and relapse prevention. Third, because the TUMS specifically focuses on strategies for coping with smoking urges, it provides a useful tool for guiding clinical practice. For example, clinicians can assess whether clients are familiar with the range of coping strategies tapped by the TUMS and educate them about unfamiliar strategies. Smokers can easily remember and tailor an appropriate response to the urge-eliciting situation (e.g., delay, escape, avoid, distract, substitute). Measures emphasizing general coping strategies e.g., [26] would be difficult to translate to a smoking urge situation. Fourth, the TUMS proved reliable and valid in a predominantly low-income and racial minority population. Relative to more affluent and non-minority populations, low-income and racial minority smokers are less likely to effectively use smoking cessation interventions [51,52] and maintain abstinence after quitting [53]. The TUMS could be a valuable tool for monitoring progress in building skills to resist smoking urges in such high-risk populations.

This study also has limitations. First, while the research sample is a strength, in that low-income smokers are under-represented, it also is a limitation. The TUMS was initially developed for use in the KiSS study. Due to the target population being a younger, primarily female, low-income, minority population, this analysis represents a relatively small, homogenous subgroup of the general population. The TUMS should be tested in a more diverse population. Second, the KiSS did not include an alternate measure of urge coping to validate the construct further. Being mindful of the demands of participating in a complex trial, we tried to keep participant measurement to a minimum.

## 5. Conclusions

Overall, this study supports a one-factor model as the best characterization of the TUMS 12-items and underlying latent factor, tobacco urge management. As a 12-item measure, the participant burden of this scale during assessments is low. Determining coping strategies as a single, overarching factor recognizes that the general application of any coping skills included in the TUMS could contribute to smoking urge management. More importantly, the one-factor model has implications for developing and implementing behavioral interventions. The TUMS can be a tool for testing treatment adherence during an intervention and measuring the change in the uptake of coping skills to manage urges during smoking cessation or relapse prevention. Overall, the findings support measuring the frequency of urge-coping strategies as a valuable assessment to understand better and promote smoking behavior change.

## Figures and Tables

**Table 1:** Tobacco Urge Management Scale (TUMS) items and factor loadings.

Item	Standardized Factor Loading
1. I mentally distracted myself from my craving. For example, I meditated, prayed, or listened to music.	0.578
2. I reminded myself of my goals for a healthier lifestyle for my family and myself.	0.761
3. I reminded myself about the negative effects of smoking when I had a craving.	0.681
4. When I had an urge during the last week, I told myself the urge would pass and that smoking was not an option.	0.704
5. In the last week, I escaped situations that made me crave a cigarette—I left high-risk situations.	0.708
6. I avoided situations or substances that I knew would increase my urge to smoke. For example, I avoided other smokers, coffee, or alcohol.	0.654
7. When I had the urge to smoke, I delayed smoking to allow myself to practice healthy coping strategies. *	0.712
8. I substituted smoking with something else, such as food or sugarless candy, when I felt the urge to smoke.	0.596
9. In the last week, I sought support to keep myself from smoking, such as calling a friend. *	0.608
10. I rewarded myself for getting through an urge or urges without smoking. *	0.721
11. I physically engaged in activities to distract myself from my craving (e.g., kept my hands busy with an activity, got up, and took a walk).	0.740
12. I engaged in relaxation or deep breathing exercises to help me manage my urge to smoke	0.642

Notes. Items 1–4 are cognitive coping strategies. Items 5–12 are behavioral coping strategies. * Three items the authors created based on theory and elements of the behavioral counseling intervention rather than O’Connell’s [40] categories. All factor loadings are significant at *p* < 0.001.

**Table 2:** Demographic and smoking characteristics of participants at baseline (*n* = 327).

Variable	*n*	%
Age (years) at baseline		
18–34	208	63.6
35–44	82	25.1
45+	37	11.3
Gender		
Men	54	16.5
Women	273	83.5
Race		
Black or African American	272	83.2
White or Caucasian	18	5.5
American Indian or Alaska Native	2	0.6
More than one race	18	5.5
Other	17	5.2
Highest education level achieved		
Some high school or less	89	27.2
High school graduate or GED	128	39.1
Vocational school or some college	94	28.7
College degree	16	4.9
Married or living with a partner		
No	193	59.0
Yes	134	41.0
Average cigarettes smoked per day		
≤10	153	46.8
>10	174	53.2

**Table 3:** Descriptive and correlational data on major variables *(n* = 298).

	1.	2.	3.	4.	5.	M	SD
1. TUMS ^a^						2.59	0.72
2. Cigarettes smoked per day ^a^	−0.34 *					4.73	4.90
3. Nonsmoking days ^b^	0.32 *	−0.75 *				13.41	21.78
4. Smoking cessation self-efficacy ^a^	0.37 *	−0.60 *	0.52 *			29.75	10.26
5. Smoke-free home/car self-efficacy ^a^	0.27 *	−0.37 *	−0.31 *	0.37 *		10.11	1.92

Notes. M = mean, SD = standard deviation, ^a^ = end-of-treatment measurement period, ^b^ = between baseline and end-of-treatment measurement period, * *p* ≤ 0.01 level (2-tailed).

## Data Availability

The data are not publicly available.
